# ROS-responsive ADPH nanoparticles for image-guided surgery

**DOI:** 10.3389/fchem.2023.1121957

**Published:** 2023-02-08

**Authors:** Kangjun Sun, Ruitong Xu, Bingyan Xue, Pengfei Liu, Jianan Bai, Ye Tian, Xiaolin Li, Qiyun Tang

**Affiliations:** ^1^ Nanjing Medical University, Nanjing, China; ^2^ Department of Geriatric Gastroenterology, The First Affiliated Hospital of Nanjing Medical University, Nanjing, China; ^3^ Department of Gastroenterology, Jiangyin People’s Hospital, Jiangyin, China

**Keywords:** nanoparticle, reactive oxygen species, image-guided, surgery, breast cancer

## Abstract

In recent years, organic fluorescent probes with tumor microenvironment (TME)-responsive fluorescence turn-on properties have been increasingly used in imaging-guided tumor resection due to their higher signal-to-noise ratio for tumor imaging compared to non-responsive fluorescent probes. However, although researchers have developed many organic fluorescent nanoprobes responsive to pH, GSH, and other TME, few probes that respond to high levels of reactive oxygen species (ROS) in the TME have been reported in imaging-guided surgery applications. In this work, we prepared Amplex^®^ Red (ADHP) with excellent ROS response performance as an ROS-responsive nanoprobe and studied its application in image-guided tumor resection for the first time. To confirm whether the nanoprobe can be used as an effective biological indicator to distinguish tumor sites, we first detected 4T1 cells with the ADHP nanoprobe, demonstrating that the probe can utilize ROS in tumor cells for responsive real-time imaging. Furthermore, we conducted fluorescence imaging *in vivo* in 4T1 tumor-bearing mice, and the ADHP probe can rapidly oxidize to form resorufin in response to ROS, which can effectively reduce the background fluorescence signal compared with the single resorufin probe. Finally, we successfully carried out image-guided surgery of 4T1 abdominal tumors under the guidance of fluorescence signals. This work provides a new idea for developing more TME-responsive fluorescent probes and exploring their application in image-guided surgery.

## 1 Introduction

Breast cancer is the prevalent malignancy in women worldwide, and in 2020 with an estimated 2.3 million new patients and 685,000 mortality cases (Sung et al., 2021), accounting for 30% of new female tumors in 2021 (Siegel et al., 2021). It is a serious threat to women’s health and an important cause of female death. The preferred treatment for breast cancer is surgical excision combined with radiotherapy and chemotherapy (Ma et al., 2020b). The surgical outcome and patient prognosis depend largely on the complete resection rate of the tumor. Postoperative tumor residue was associated with poor prognosis, high recurrence rate, and low survival rate. The higher the tumor resection rate is, the longer the overall survival of patients (Kimbrough et al., 2013; McCann et al., 2013; Tummala et al., 2013). At present, the extent of surgical resection still relies heavily on the surgeon’s experience. However, the visual and tactile distinction between tumor and healthy tissue is not effective, making it difficult to determine the surgical margins and leaving tiny lesions that can lead to recurrence and spread after surgery, while the quality of life of patients is seriously affected if the extent of surgical excision is excessively extended. Therefore, the key to the success of surgery is how to accurately locate and image tumors and their microscopic lesions intraoperatively and how to maximize the removal of tumors while protecting healthy tissues as much as possible. Recently, molecular imaging techniques has rapidly advancing in the field of bioimaging due to its advantages of great sensitivity, speedy and immediate imaging, biological safety, ease of detection and low cost, etc. Fluorescence imaging-guided surgery brings hope to solving the abovementioned challenges (Antaris et al., 2016; Zheng et al., 2017; Qi et al., 2018).

Molecular imaging techniques allows non-invasive real-time tumor diagnosis and imaging-guided surgery, assisting surgeons in detecting and resecting tiny tumors sensitively and accurately, significantly improving the therapeutic outcome of tumor surgery. Fluorescent probes could improve the signal-to-background ratio (SBR) by increasing the target signal or decreasing the background signal intensity to enhance the imaging sensitivity and specificity (Owens et al., 2016). Fluorescent probes can generally be divided into two categories: “Always ON” probes and “Turn ON” probes (responsive probes) (Lou et al., 2015; Liu et al., 2017; Jiao et al., 2018; Li and Pu, 2019; Zhang et al., 2019; Xu et al., 2022). “Always ON” probes emit fluorescence continuously under all conditions (whether they reach the target), which increases the background signal and reduces the SBR. In contrast, “Turn ON” probes change the fluorescence signal from “off” to “on” in response to the target (e.g., pH, ROS, or bioenzyme), maximizing the target signal while minimizing the background signal, thus maximizing the SBR, improving the sensitivity and resolution of biosensing (Tang et al., 2019).

Among them, organic fluorescent probes with the tumor microenvironment (TME)-responsive fluorescence-on properties have attracted our interest. The breast TME is a complex ecological environment. In addition to hypoxia, acidosis, elevated levels of lactic acid and adenosine (Cassim and Pouyssegur, 2020), and reactive oxygen species (ROS) levels are much higher than those in normal tissues (Xu et al., 2017; Ma et al., 2020a; Malla et al., 2021; Zhang et al., 2021). Although many organic fluorescent nanoprobes, such as pondus hydrogenii (pH), glutathione (GSH), and other TME responses, have been developed by researchers and applied to imaging-guided surgery, fluorescence probes that respond to high levels of ROS in the TME have rarely been reported. We hypothesized that high levels of ROS in the TME could be used as a bioindicator to identify solid tumors and thus use ROS-responsive fluorescent probes to accurately identify tumor tissue and perform image-guided surgery. In this work, the 10-acetyl-3, 7-dihydroxyphenoxazine (Amplex^®^ Red, ADPH) with excellent ROS responsiveness was prepared as a ROS-responsive nanoprobe, and the performance of ROS-responsive fluorescence imaging was first assessed at the cellular level. Then, its fluorescence imaging sensitivity in complex biological environment *in vivo* was validated in mice models, and its application in image-guided tumor resection was investigated. The results show that the ROS-responsive fluorescent nanoprobe can perform effective fluorescence imaging of tumors and their microscopic lesions, providing a new theoretical basis for the application of responsive fluorescent probes in the surgical navigation of breast cancer.

## 2 Experimental section

### 2.1 Materials and methods

All chemicals and reagents were acquired from chemical sources and applied as received. ADPH (Jinming Biotechnology), resorufin (Macklin), 1, 2- distearoyl-sn-glycero-3-phosphoethanolamine-N-[methoxy- (polyethylene glycol)-2000] (DSPE-PEG_2000_) (Energy Chemical), hydrogen peroxide (H_2_O_2_, Enokai Technology), 3-(4, 5-dimethylthiazol-2-yl)-2, 5-diphenyltetrazolium bromide (MTT, Sigma), horseradish peroxidase (HRP, Sigma), buthionine sulfoximine (BSO, Macklin). The murine 4T1 breast cancer cell lines were obtained from American Type Culture Collection (ATCC). The absorbance and fluorescence spectra were obtained using a PerkinElmer Lambda 365 spectrophotometer and a HITACHI F-4,700 fluorescence spectrophotometer. Investigation of dynamic light scattering (DLS) using a 90 plus particle size analyzer. The *in vivo* fluorescence imaging was taken by an IVIS Lumina II (Xenogen).

### 2.2 Preparation of NPs

A compound of fluorescent probes (1 mg), DSPE-PEG_2000_ (4 mg), and dimethyl sulfoxide (DMSO) (1 mL) were sonicated to obtain a clear solution by complete dissolution. The solution was then speedily injected into 10 mL of distilled water over 4 min using a microtip probe ultrasound generator (XL2000, Misonix Consolidated, NY). The compound was then shifted into a permeation bag (molecular weight cutoff (MWCO) = 5,000 Da), permeated in distilled water for 24 h, ultrafiltered to 1 mL by ultrafiltration (MWCO = 10,000 Da) and filtered through a 0.2 μm syringe filter before use.

### 2.3 Cell culture

The 4T1 breast cancer cell lines were grown and incubated in Dulbecco’s modified Eagle’s medium (DMEM) containing 10% fetal bovine serum and 1% penicillin/streptomycin at 37°C, 5% CO_2_, saturated humidity in a cell culture incubator.

### 2.4 *In vitro* cytotoxicity study

4T1 breast cancer cells were inoculated in 96-well plates maintaining a density of 5,000 cells per well and the MTT assay was performed after 24 h of adhesion. The cells were then incubated with various concentrations of ADHP and resorufin NPs for 24 h. Then 10 μL of freshly made MTT solution (medium concentration of 5 mg/mL) was added to each well. After a total incubation of 4 h, the supernatant was discarded and 100 μL of DMSO was added to dissolve the precipitate, which was gently shaken on a shaker. The absorbance of MTT at 490 nm was measured by an enzymatic standard (GENios Tecan). The absorbance of cells incubated with NPs was expressed as the ratio of the absorbance of cells incubated with NPs to the absorbance of cells incubated in medium only.

### 2.5 Tumor-bearing mouse model

Following the guidelines of the Tianjin Experimental Animal Use and Care Committee, the overall project protocol was approved by the Animal Ethics Committee of Nankai University. All animal studies were conducted using 6-week-old female BALB/c mice, purchased from the Experimental Animal Centre of the Chinese Academy of Military Medical Sciences. To establish the mice transplantation models for breast cancer, 4T1 cells (5 × 10^5^) were taken and mixed with 100 μL of phosphate buffered saline (PBS) and injected into the peritoneal cavity of mice. Tumors were grown for approximately 7 days followed by fluorescence imaging or surgical treatment experiments.

### 2.6 *In vivo* fluorescence imaging

Fluorescence imaging of ADHP NPs and resorufin NPs in 4T1 subcutaneous tumor-bearing mice was performed. Mice were intravenous with ADHP NPs or resorufin NPs (200 μL, 30 μM based on ADHP or resorufin) and sacrificed after 24 h, and the major organs (heart, liver, spleen, lungs, kidneys, tumours) were obtained and imaged using IVIS Lumina II.

### 2.7 Fluorescence imaging-guided tumor surgery

The mice models of peritoneal metastasis of luciferase-expressing 4T1 were intravenous with 200 μL of ADHP NPs (30 μM based on ADHP). 24 h later, the mice were anesthetized with 2% isoflurane, the abdominal cavities were dissected, bioluminescence imaging and fluorescence imaging of the peritoneal metastases were performed, and the metastases and residual microscopic tumor nodules were excised following fluorescence imaging guidance.

### 2.8 Histological study

Histological analysis was carried out on the tumors excised in the aforementioned fluorescence guided-image surgery. Simply, tumors were immobilized in 4% paraformaldehyde, embedded into wax blocks, and sectioned to a thickness of 5 μm, followed by hematoxylin-eosin (H&E) staining. Pathological sections were ured by a digital microscope (Leica QWin).

## 3 Results and discussion

### 3.1 Photophysical properties of molecules

**FIGURE 1:**
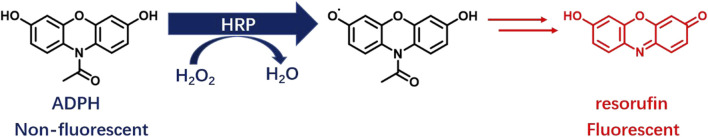
Scheme of the oxidative transformation of ADPH to resorufin.

Mammalian cells can produce ROS through a variety of mechanisms, of which H_2_O_2_ is one of the main types and plays various essential roles in cellular physiological and pathological processes (Cheung and Vousden, 2022). ADPH, catalyzed by HRP, can react with ROS (mainly H_2_O_2_) to produce resorufin, which has red fluorescence (Zhou et al., 1997) (Figure 1).

**FIGURE 2:**
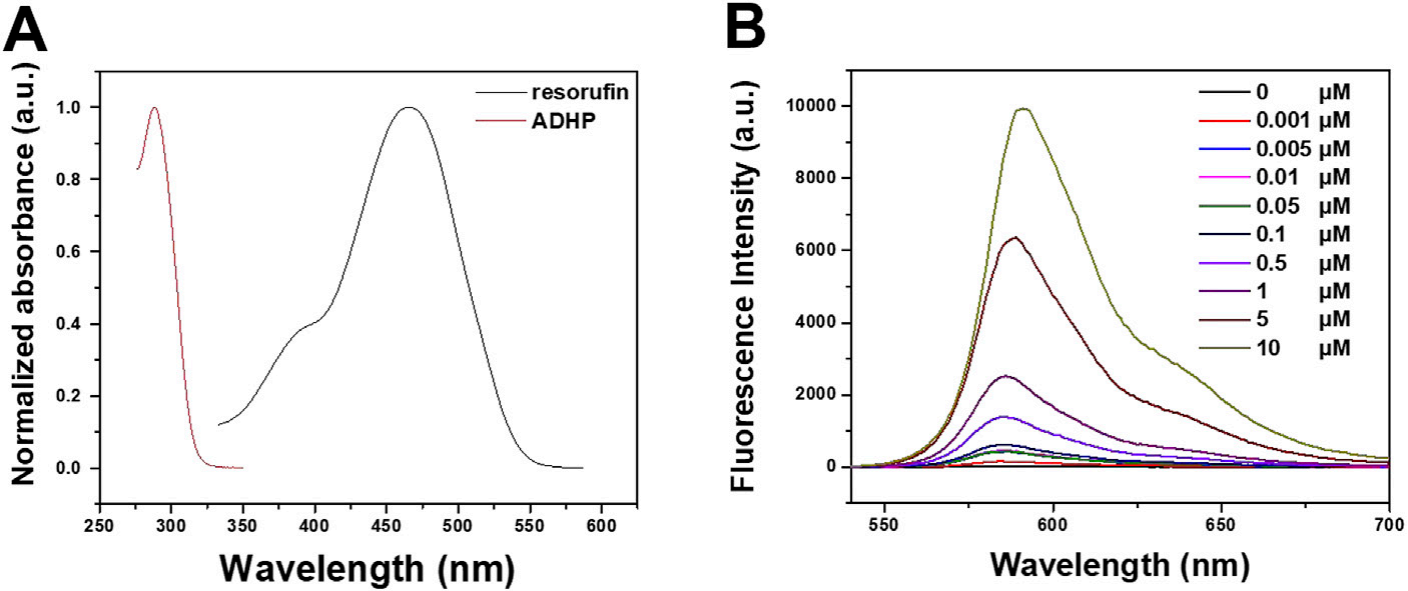
**(A)** Absorption spectra of ADHP and resorufin in DMOS. **(B)** Fluorescence intensity variation of ADPH in response to different concentrations of H_2_O_2_.

The photophysical properties of ADPH and resorufin in DMSO were first investigated. Figure 2A showed the absorption spectra of ADPH in DMSO with a peak at 288 nm, then resorufin with an absorption peak at 467 nm and an emission peak at 570 nm (Supplementary Figure S1). Further the fluorescence intensity variation of ADPH in response to various concentrations of H_2_O_2_ was studied. The results showed that different concentrations of H_2_O_2_ (from 0 to 10 μM) reacted with 2 μM ADPH under 0.1 U/mL HRP catalysis, resulting in a concentration-dependent increase in the fluorescence emission intensity of the reaction product at 590 nm (Figure 2B). These results indicate that ADPH can effectively respond to ROS stimulation and turn on the fluorescence signal, which provides the possibility of a responsive tracer.

### 3.2 Photophysical properties of nanoparticles (NPs)

**FIGURE 3:**
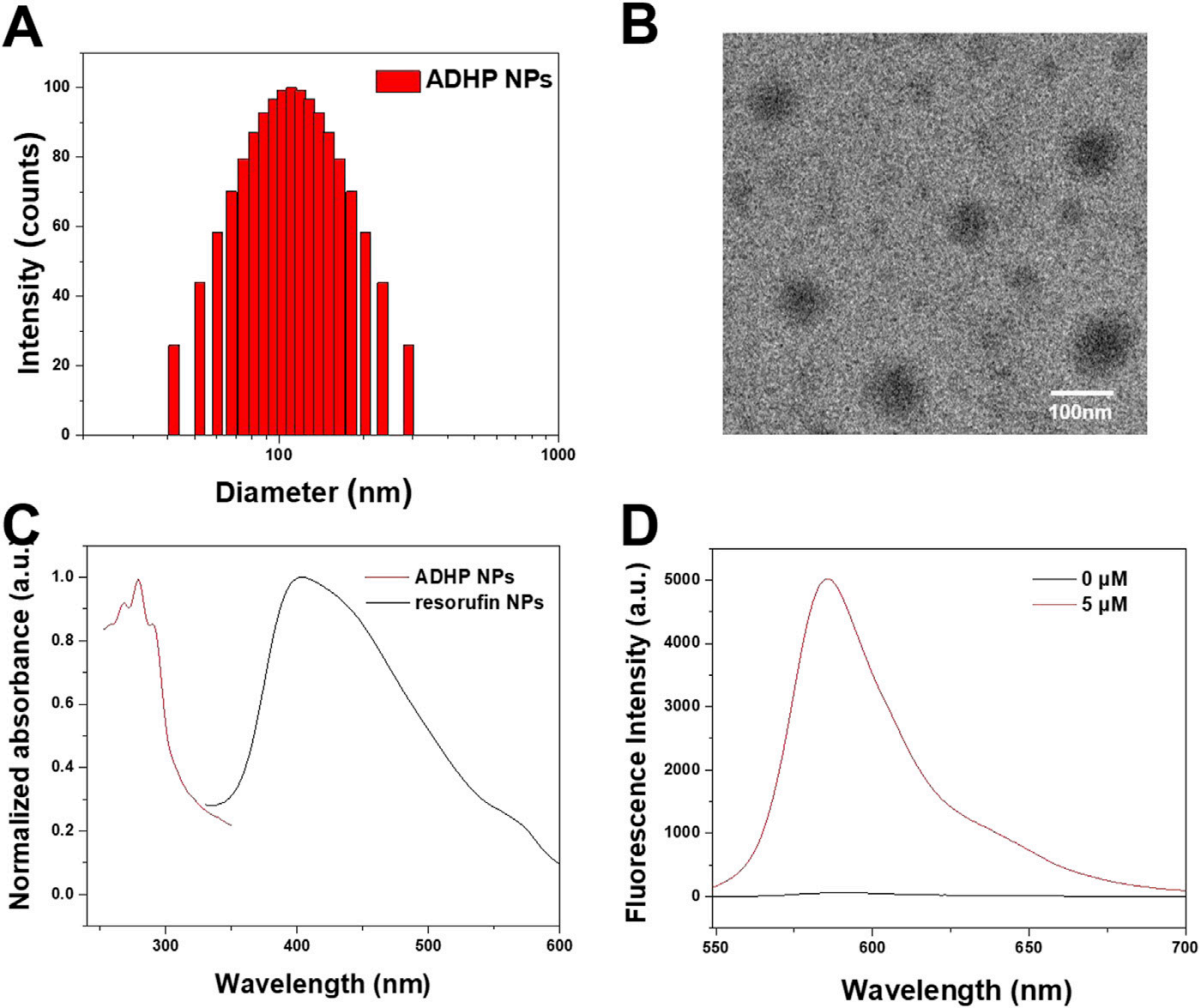
**(A)** DLS profile of ADHP NPs. **(B)** Representative transmission electron microscopy images of the ADHP NPs. **(C)** Absorbance spectra of ADHP NPs and resorufin NPs. **(D)** Fluorescence spectra of ADHP NPs (concentration 0 and 5 μM) after responding with H_2_O_2_.

To increase the aqueous dispersion of ADHP and resorufin molecules, a biocompatible amphiphilic polymer, DSPE-PEG_2000_, was used as an encapsulation matrix to encapsulate the hydrophobic pores of NPs by nanoprecipitation (Li et al., 2018). The ADHP NPs and resorufin NPs could be uniformly dispersed in water to form pale-red and orange–red solutions with high transparency, respectively. The DLS outcomes indicated that the average hydrodynamic diameters of the ADHP NPs and resorufin NPs were 110.7 nm and 141.43 nm, respectively (Figure 3A; Supplementary Figure S2A), which allowed the NPs to passively target tumor tissue with enhanced permeation and retention (EPR) effects (Cheng et al., 2013). The morphology of the ADHP NPs was characterized by transmission electron microscopy (TEM), showing a homogeneous spherical structure with an average diameter of about 110 nm (Figure 3B). The slightly larger diameter measured by DLS relative to the TEM results may be due to the shrinkage of nanoparticles during TEM sample preparation. Both NPs and resorufin NPs showed good colloidal stability in 10% serum aqueous solution (Supplementary Figure S3), and the nanoprobe solution remained clear and transparent for a week. Furthermore, the UV absorption and fluorescence spectra of the nanoprobes were investigated in an aqueous solution. The maximum absorption peak of ADHP NPs was located at 281 nm, and the emission peak was located at 585 nm after responding with H_2_O_2_ (Figures 3C, D). The maximum absorption and emission peaks of resorufin NPs were located at 404 nm (Figure 3C) and 594 nm (Supplementary Figure S2B), respectively.

### 3.3 Biocompatibility of NPs

**FIGURE 4:**
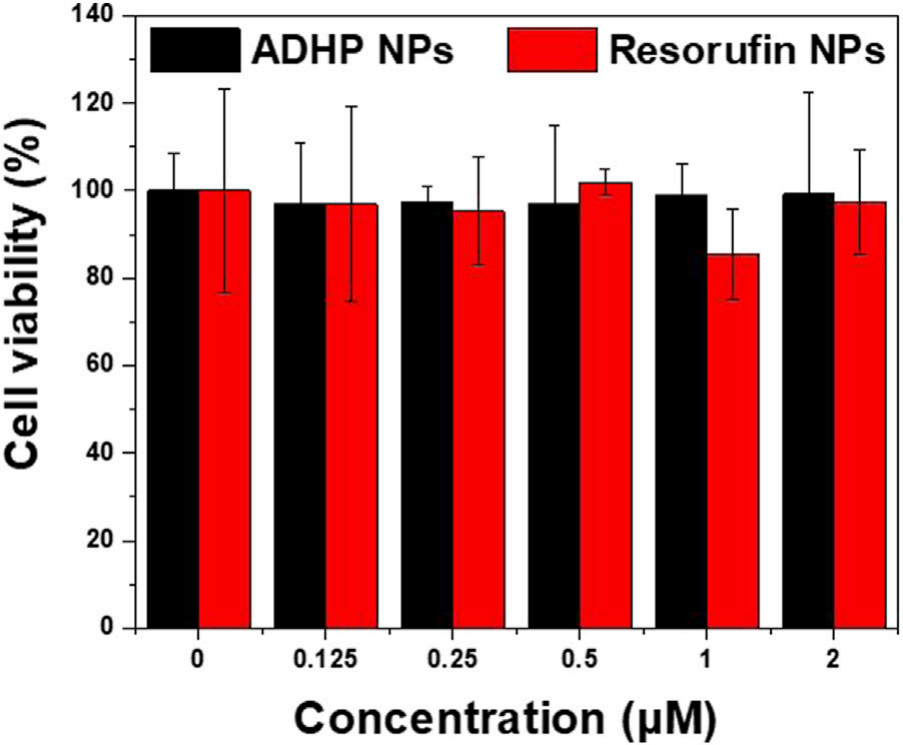
The viability of 4T1 cells after incubation with various concentrations of ADHP NPs and resorufin NPs protected from light for 24 h.

Biocompatibility is key to the application of nanoprobes. Prior to the *in vivo* study, the cytotoxicity of ADHP NPs and resorufin NPs *in vitro* were first examined. As shown in Figure 4, after coincubation with 4T1 breast cancer cell lines for 24 h with different concentrations of NPs, the cell survival rate was higher than 95% in each group, and no significant cytotoxicity was observed. The cellular uptake of the nanoprobes was subsequently examined. Confocal microscopy showed that resorufin NPs could be effectively internalized and localized in the cytoplasm after coincubation with 4T1 cells for 4 h (Supplementary Figure S4). These results suggest that the NPs are biocompatible and can be effectively taken up by breast cancer cells.

### 3.4 Fluorescence imaging of ADHP NPs *in vitro*

**FIGURE 5:**
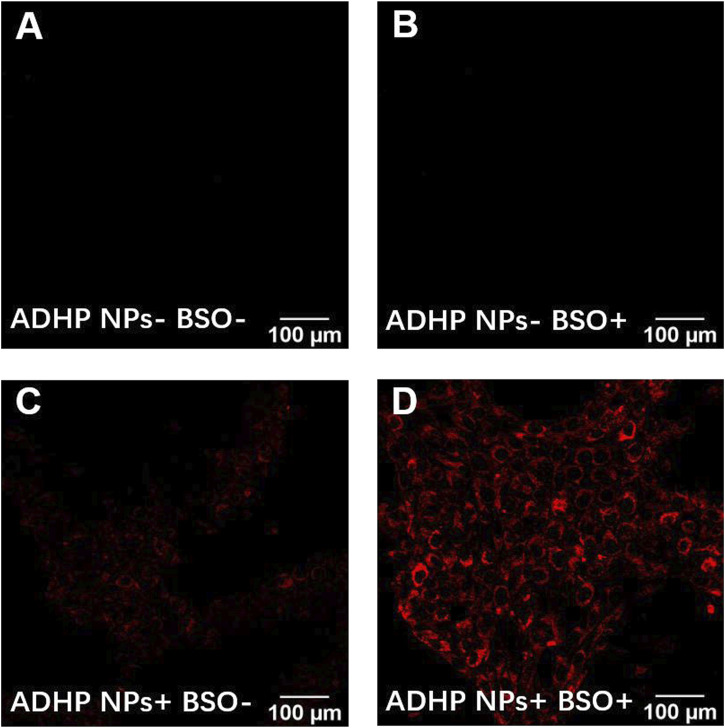
Imaging under fluorescence microscope of ADHP NPs *in vitro*: 4T1 cells **(A)** incubated with PBS for 7 h, **(B)** preincubated with PBS for 4 h, later were exposed to BSO (50 mM) for another 3 h, **(C)** incubated with ADHP NPs(2 μM) for 7 h, **(D)**preincubated with ADHP NPs (2 μM) for 4 h, later were added BSO (50 mM) and incubated for another 3 h.

ROS play a crucial role in the breast TME and are associated with a range of pathophysiological processes, including regulating cell proliferation, activating oncogenes, mediating genomic instability, inducing inflammation, initiating metabolic reprogramming, and promoting metastasis (Kalyanaraman et al., 2018; Weinberg et al., 2019). Many studies have confirmed that ROS levels in the breast TME are higher than those in normal tissues (Malla et al., 2021). To validate the ROS response of ADHP NPs and the ability to image cells *in vitro*, the BSO, a glutamylcysteine synthase inhibitor, was used to increase intracellular H_2_O_2_ levels. Confocal fluorescence microscopy results showed that in the two groups without the addition of ADHP NPs, no fluorescence signal was detected in the group with or without BSO (Figures 5A, B). In the other two groups, after co-incubation with ADHP NPs for 4 h, one group added 50 mM BSO and incubated for another 3 h. As shown in Figure 5C, there was a weak fluorescence signal in the BSO (−) group, indicating a low level of ROS in 4T1 cells cultured *in vitro*, while the fluorescence brightness in the BSO (+) group was significantly enhanced (Figure 5D). It is suggested that the addition of BSO can significantly increase the level of ROS in tumor cells, and ADHP NPs as a responsive fluorescent probe can fully respond and turn on the fluorescent signal for imaging.

### 3.5 Fluorescence imaging of tumors

**FIGURE 6:**
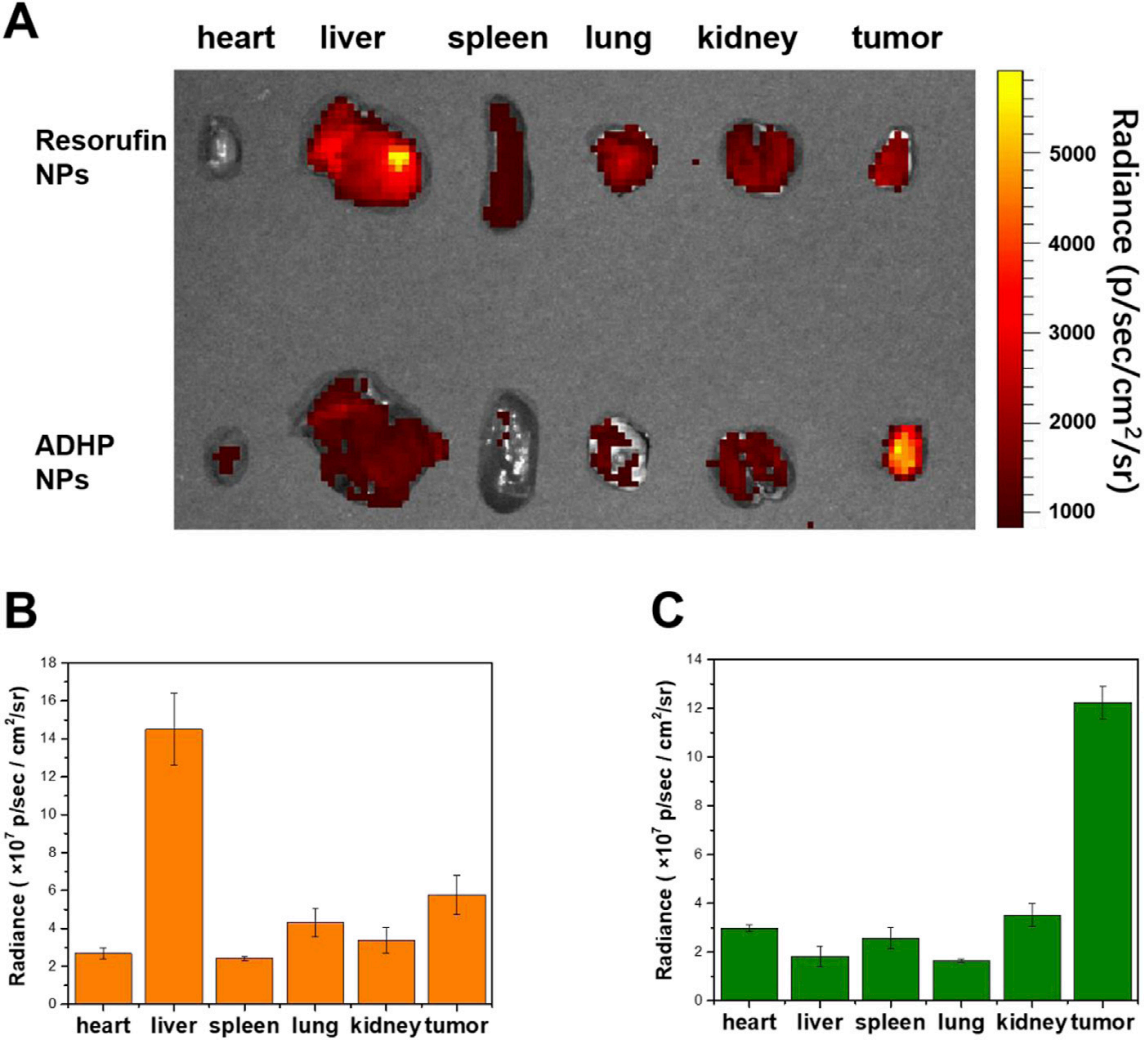
**(A)** *Ex vivo* fluorescence imaging of separate tissues of 4T1 subcutaneous tumor-bearing mice injected intravenously of 200 μL of ADHP NPs or resorufin NPs (30 μM based on each molecule) for 24 h. **(B)** Mean fluorescent signals of dissimilar tissues based on the images in the resorufin NPs group. **(C)** The average fluorescent signals in dissimilar tissues based on the images in the ADHP NPs group.

*In vitro* experiments confirmed that ADHP NPs can respond to ROS in tumor cells to switch on the fluorescent signal; therefore, the *in vivo* performance of ROS-responsive fluorescent probes for fluorescence imaging in tumor-bearing mice was further assessed by calculating the tumor-liver signal ratio. ADHP NPs and resorufin NPs were intravenously injected into 4T1 subcutaneous tumor-bearing mice, respectively. The mice were sacrificed 24 h later, and the tumors and major organs were removed for fluorescence imaging at the same time (Figure 6A). *Ex vivo* fluorescence imaging showed that the liver, lung, and tumor in the resorufin NPs group displayed fluorescence, of which the liver had the strongest fluorescence intensity. The fluorescence ratio of the tumor to the liver was only 0.4 (Figure 6B), suggesting that the tumor-to-liver signal ratio of the “Always ON” fluorescent probe was low. Due to the strong liver fluorescence signal background during *in vivo* imaging, it is difficult to distinguish the tumor fluorescence signal. In contrast, in the ADHP NPs group, although the liver, lung, and tumor showed fluorescence signals, the fluorescence ratio of the tumor to the liver was as high as 6.67 (Figure 6C). These results suggest that because of the higher ROS levels in tumor tissue than in normal tissue, ROS-responsive ADHP NPs can respond adequately to them and switch on the fluorescent signal. Thus, the interference of background signals in fluorescence imaging can be minimized. More importantly, the fluorescence signal of the liver is negligible, which improves the sensitivity and resolution of fluorescence imaging and shows great advantages in precise image-guided tumor surgery.

### 3.6 Fluorescence imaging-guided tumor surgery

**FIGURE 7:**
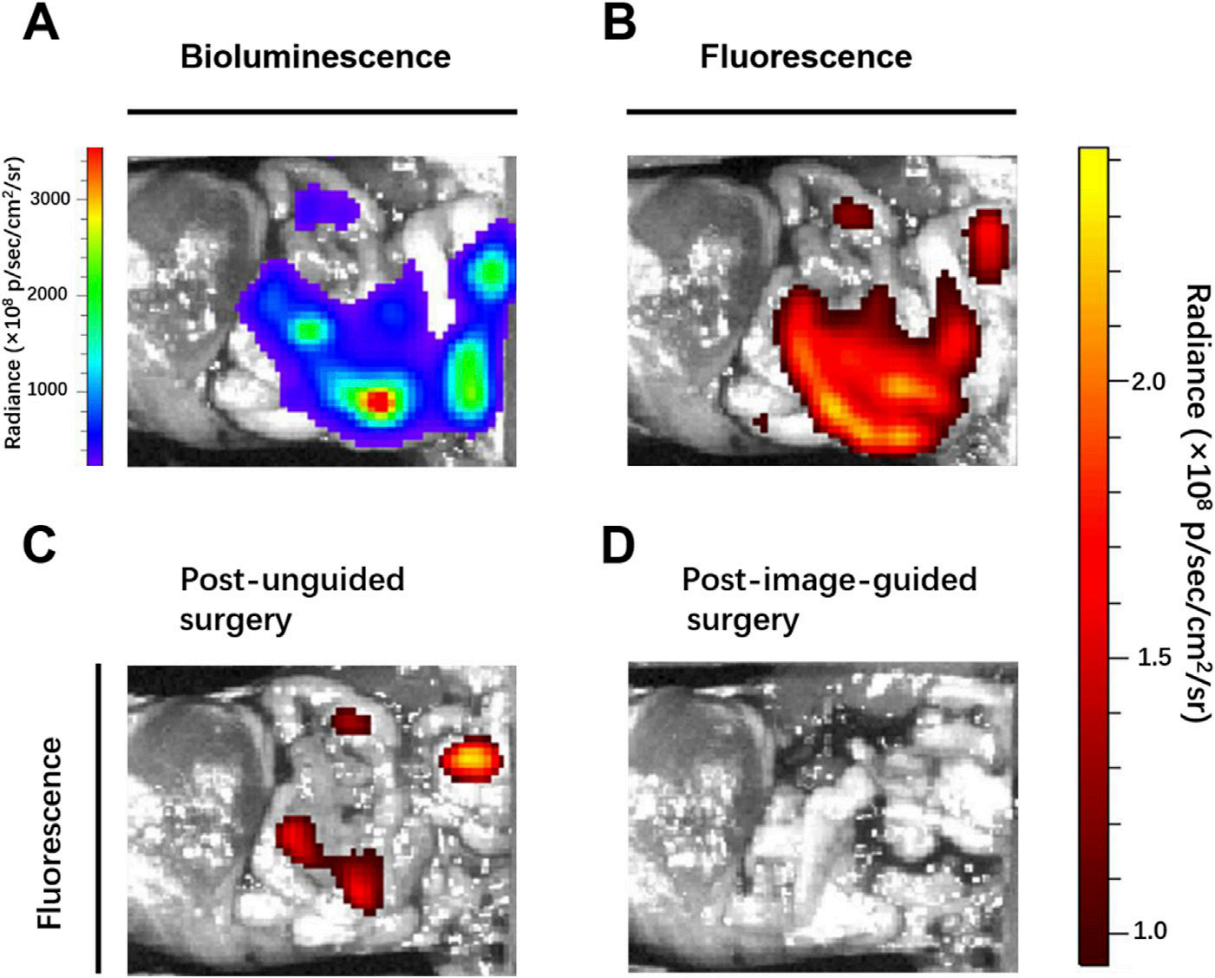
Mice with peritoneal metastases were intravenously injected with 200 μL of ADPH NPs (30 μM based on ADHP) and operated on 24 h after injection: **(A)** bioluminescence and **(B)** fluorescence images of the peritoneal cavity of mice before tumor resection. Fluorescent images typical of mice **(C)** post unguided surgery and **(D)** post second surgery with guidance of the fluorescence of ADHP NPs.

Breast cancer is the commonest malignancy in women and is prone to distant metastases in the lung, liver, bone and brain, and is a major cause of death (Ahmad, 2019; Slamon et al., 2019). Patients with distant metastases have a poor prognosis, the five-year survival rate was only 27% (DeSantis et al., 2019). In clinical surgical oncology, intraoperative imaging to precisely locate tumor nodes, detect and completely remove all tumor lesions can greatly improve the success rate of surgery and avoid tumor recurrence. After abdominal metastasis of breast cancer, many tiny tumor nodules are scattered in the peritoneal cavity, leading to further metastasis, and spread of the tumor, and the survival rate of patients is significantly reduced. Therefore, accurate preoperative evaluation and intraoperative real-time imaging are necessary. Thus, we established mice models of peritoneal metastasis of 4T1 breast cancer cells to assess the ability of ADHP NPs as an ROS-responsive nanoprobe to identify microscopic tumor nodules *in vivo*. To detect the tumor distribution, we selected luciferase-expressing 4T1 breast cancer cell lines, which showed bioluminescence after injection of luciferin. After intravenously injecting ADHP NPs (200 μL, 30 μM based on ADHP) into a mouse for 24 h, the mouse’s abdominal cavity was dissected for bioluminescence imaging and fluorescence imaging. As a result of the EPR effect of tumors (Cheng et al., 2013), ADPH NPs can be enriched in the tumor site and the fluorescence signal of ADHP NPs completely coincided with the bioluminescence signal of fluorescein in the peritoneal cavity. It is proved that ADHP NPs can turn on the fluorescence signal in response to the high concentration of ROS in TME and accurately locate the tumor lesions. (Figures 7A, B).

**FIGURE 8:**
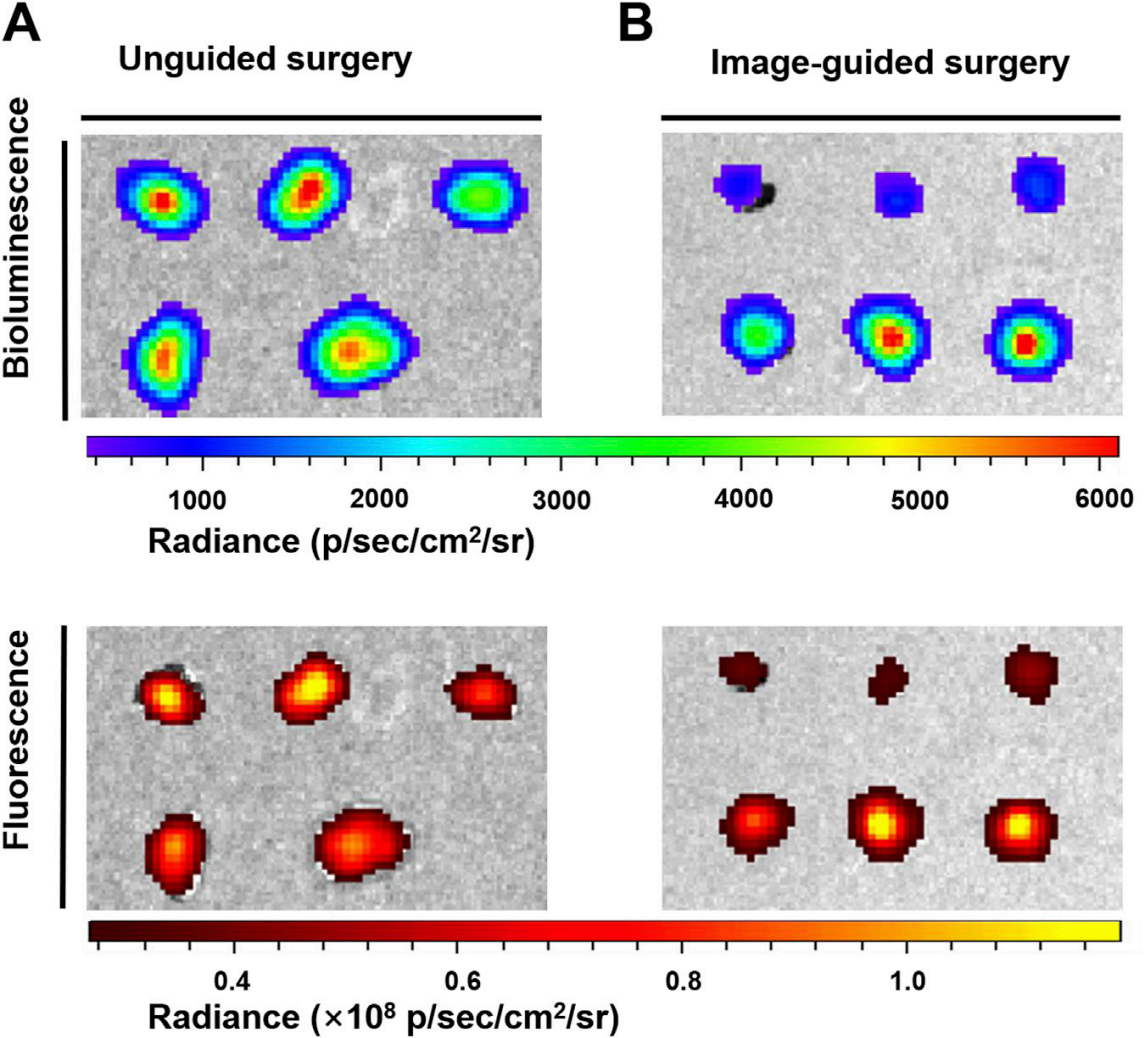
The peritoneal carcinomatosis-bearing mice were intravenously injected with 200 μL of ADPH NPs (30 μM based on ADHP) followed by the operation of the abdomen at 24 h after injection: bioluminescence and fluorescence images typical of resected tumor nodules **(A)** post unguided surgery and **(B)** post the second surgery with guidance of fluorescence imaging of ADPH NPs.

Clinically, surgeons mainly rely on the naked eye to distinguish which tissues need to be removed and retained. Although sizeable tumors (>1 mm) were excised by the surgeon (the First Affiliated Hospital of Nanjing Medical University) after naked eye resolution, there were still some tiny (submillimeter level) unidentifiable tumor nodules remaining. With the help of the fluorescence signal of the high SBR of AHDP NPs, the residual tiny metastases in the peritoneal cavity of the tumor-bearing mice were clearly visible. Therefore, a second surgical resection was performed under the guidance of fluorescence imaging signals, and the tumors were observed to be excised postoperatively (Figures 7C, D). The operation took approximately 15 min. The bioluminescence signals and fluorescence signals of all resected tumor nodules completely overlapped (Figures 8A, B). Histological staining confirmed that the resected tissues were tumors (Supplementary Figure S5). In this study, by ROS responsive fluorescence signal of ADHP NPs, we maximized the fluorescence signal intensity in tumor tissue, while minimizing the background fluorescence signal intensity, providing high resolution real-time fluorescence images about the tumor tissue during surgery, improving the detection rate of microscopic lesions, assisting surgeons in pinpointing tumor lesions, improving the efficacy of surgical resection, and greatly reducing the risk of tumor recurrence.

## 4 Conclusion

In conclusion, we prepared a ROS-responsive fluorescent probe based on ADHP, which has ROS-responsive properties. *In vitro* studies have shown that ADHP NPs can effectively respond to ROS in tumors to turn on the fluorescent signal for imaging and have good biocompatibility. Using resorufin NPs as the contrast agent, the tumor-to-liver signal ratio of ADHP NPs was first measured *in vivo*, demonstrating that ROS-responsive fluorescent probes can effectively reduce the background signal interference in fluorescence imaging. Subsequently, under the real-time fluorescence signals guidance of ADHP NPs, peritoneal metastases and their boundaries with normal tissues were clearly displayed, and the tiny tumor lesions were accurately located and resected. The ROS-responsive fluorescence probes can minimize the background signal in fluorescence imaging and provide accurate intraoperative visualization of submillimeter tumor lesions with higher sensitivity and resolution, ensuring complete tumor removal. The ROS-responsive fluorescence probe offers more possibilities for the future use of responsive fluorescence probes in surgical navigation.

## Data Availability

The original contributions presented in the study are included in the article/Supplementary Material, further inquiries can be directed to the corresponding author.
