# Supplementary material for: ROS-responsive ADPH nanoparticles for image-guided surgery
Source: Front Chem. 2023 Feb 8;11:1121957. doi: 10.3389/fchem.2023.1121957 (PMC9944124; doi:10.3389/fchem.2023.1121957)
Supplement: Supplementary file 1 [file DataSheet1.docx]

**Supporting information**


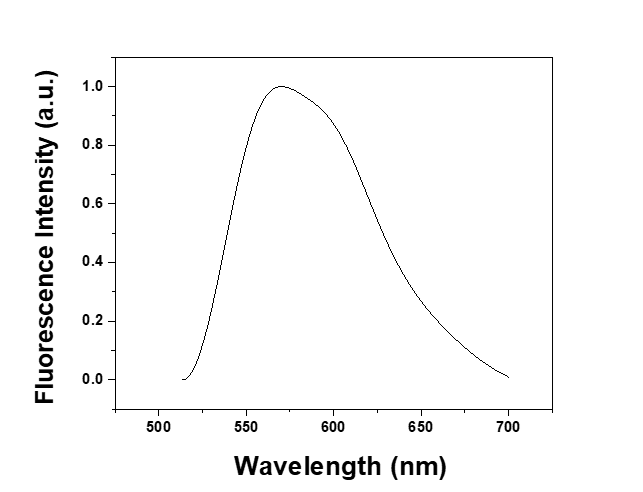


Figure S1. Fluorescence spectra of resorufin in DMSO.


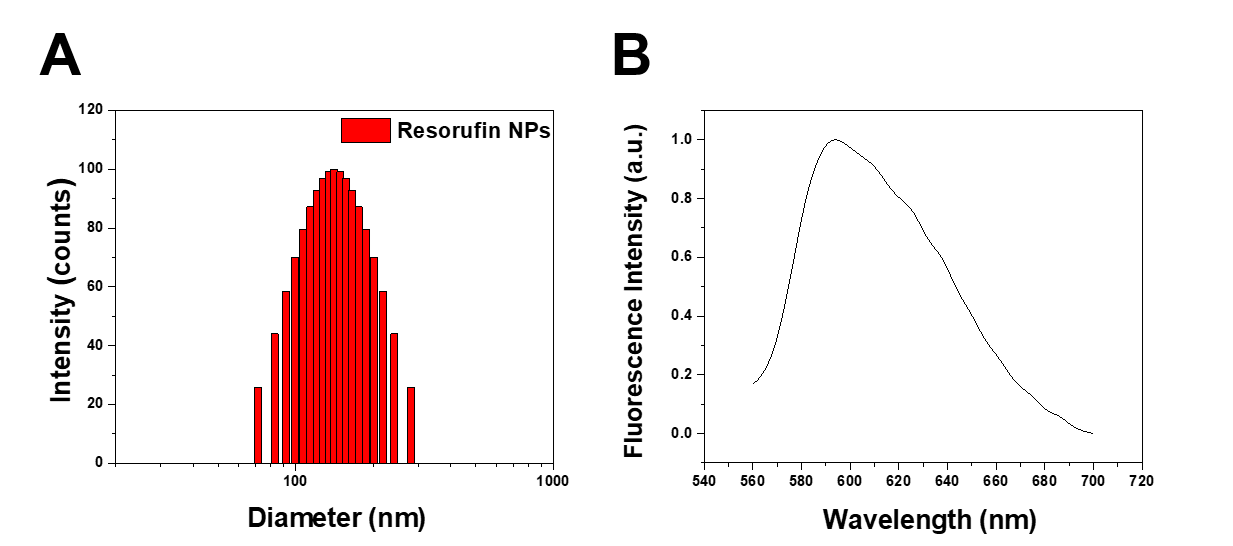


Figure S2. (A) DLS profile of resorufin NPs. (B) Fluorescence spectra of resorufin NPs.


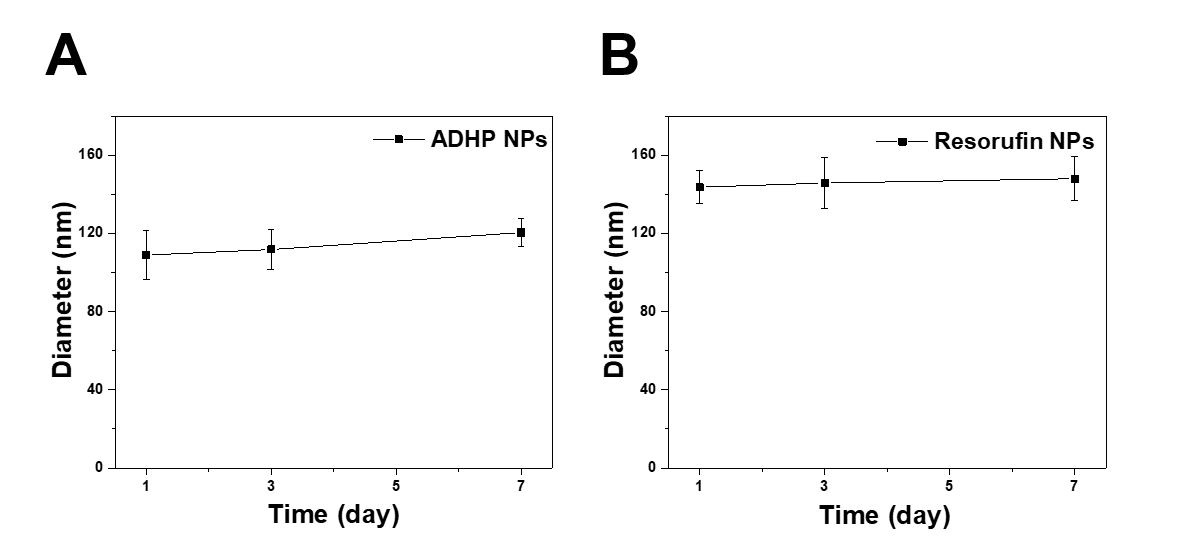


Figure S3. Colloid stability of ADHP NPs (A) and resorufin NPs (B) in 10% serum aqueous solution over time.


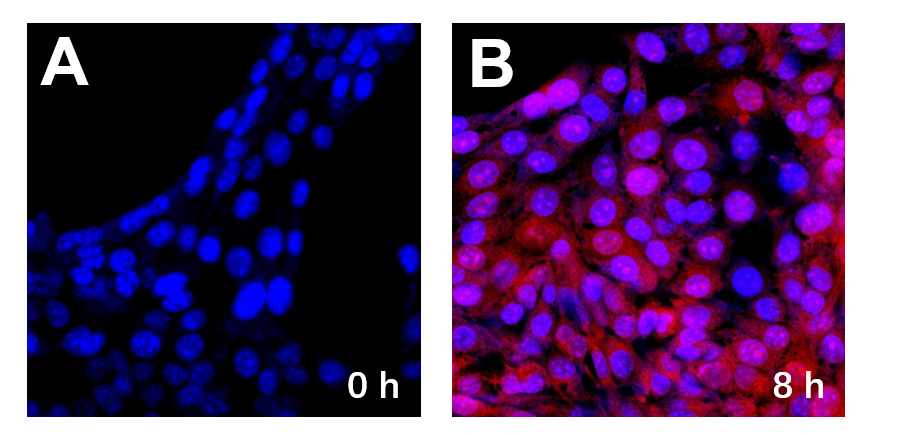


Figure S4. Confocal images of resorufin NPs uptake by 4T1 breast cancer cells.


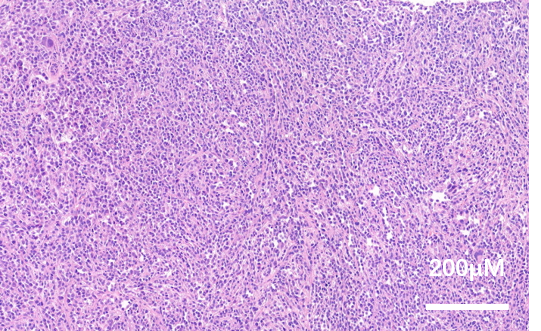
.

Figure S5. H&E staining of excised tumor.
